# Overcoming the Inflammatory Stage of Non-Healing Wounds: In Vitro Mechanism of Action of Negatively Charged Microspheres (NCMs)

**DOI:** 10.3390/nano10061108

**Published:** 2020-06-03

**Authors:** Edorta Santos-Vizcaino, Aiala Salvador, Claudia Vairo, Manoli Igartua, Rosa Maria Hernandez, Luis Correa, Silvia Villullas, Garazi Gainza

**Affiliations:** 1 NanoBioCel Group, Laboratory of Pharmaceutics, University of the Basque Country (UPV/EHU), School of Pharmacy, Paseo de la Universidad 7, 01006 Vitoria-Gasteiz, Spain; edorta.santos@ehu.eus (E.S.-V.); aiala.salvador@ehu.eus (A.S.); manoli.igartua@ehu.eus (M.I.); rosa.hernandez@ehu.eus (R.M.H.); 2 Biomedical Research Networking Center in Bioengineering, Biomaterials and Nanomedicine (CIBER-BBN), 01006 Vitoria-Gasteiz, Spain; 3 BioKeralty Research Institute AIE, Albert Einstein, 25-E3, 01510 Miñano, Spain; silvia.villullas@keralty.com; 4 Praxis Pharmaceutical, San Fernando de Henares Business Park, Avenida de Castilla 2, Dublin Building 2nd floor, San Fernando de Henares, 28830 Madrid, Spain; lcorrea@praxisph.com

**Keywords:** chronic wound, device, foot ulcer, inflammation, wound healing, macrophage

## Abstract

Negatively charged microspheres (NCMs) represent a new therapeutic approach for wound healing since recent clinical trials have shown NCM efficacy in the recovery of hard-to-heal wounds that tend to stay in the inflammatory phase, unlocking the healing process. The aim of this study was to elucidate the NCM mechanism of action. NCMs were extracted from a commercial microsphere formulation (PolyHeal^®^ Micro) and cytotoxicity, attachment, proliferation and viability assays were performed in keratinocytes and dermal fibroblasts, while macrophages were used for the phagocytosis and polarization assays. We demonstrated that cells tend to attach to the microsphere surface, and that NCMs are biocompatible and promote cell proliferation at specific concentrations (50 and 10 NCM/cell) by a minimum of 3 fold compared to the control group. Furthermore, NCM internalization by macrophages seemed to drive these cells to a noninflammatory condition, as demonstrated by the over-expression of CD206 and the under-expression of CD64, M2 and M1 markers, respectively. NCMs are an effective approach for reverting the chronic inflammatory state of stagnant wounds (such as diabetic wounds) and thus for improving wound healing.

## 1. Introduction

Hard-to-heal and chronic wounds are those that are not able to adequately continue the healing process, prolonging the situation for longer than 3 months [1,2]. The cost derived from chronic wounds in the US is estimated at more than 25 billion dollars a year, affecting more than 6 million people. Approximately 1% of the population of developed countries will suffer these injuries at some point in their lives [3]. Especially important are those known as diabetic foot ulcers, affecting 15% of diabetic patients, which in the US alone are around 20 million and expected to double by 2030 [4].

In this kind of wound, the healing process is incomplete and interrupted due to multiple factors. Senescent fibroblasts exhibit a decreased migration and synthesis of collagen, accompanied by high protease activity, which inhibits important factors in the healing process (e.g., platelet-derived growth factor (PDGF), transforming growth factor beta (TGF-β) or vascular endothelial growth factor (VEGF)) [5], and keratinocytes become senescent and unable to migrate and close the wound [6].

During the healing process, the macrophage population skews from a predominant pro-inflammatory (M1) to an anti-inflammatory (M2) phenotype, releasing anti-inflammatory mediators and growth factors [7,8]. Moreover, they can generate precursors for fibroblast activation and collagen synthesis [9] which is necessary for suitable wound healing [10]. However, chronic wounds persist in the inflammatory state, with M1 macrophages predominating [11].

An interesting option for the treatment of this type of wound consists of strategies that promote proliferation of the main cells involved in wound healing (keratinocytes and fibroblasts) and allows pro-inflammatory macrophages to skew to an anti-inflammatory phenotype. For that purpose, biomaterials can play an important role. Many alternatives have already been explored in this sense, such as liposomes, nanoparticles, microparticles or scaffolds [12]. Indeed, the type and level of cytokines produced by biomaterial-adherent cells can be modulated in order to regulate the immunologic response, with hydrophilic and anionic surfaces being among the most efficient in preventing inflammatory responses [13].

Negatively charged microspheres (NCMs) are a novel example of microtechnology used in the field of regenerative medicine. These synthetic biocompatible particles present unique biophysical properties, which make NCMs a valuable biomaterial for treating hard-to-heal wounds of different etiologies [14]. Furthermore, NCMs have been recently employed in wound healing for their excellent clinical results, i.e., in nonresponding diabetic foot ulcers [15] and wound dehiscence following breast reduction or mastopexy surgery [16]. Their size (~5 µm) and negative surface charge (zeta potential ~ −40 mV) allow the attachment and migration of cells involved in the healing process, ultimately improving wound healing [17]. Particularly, NCMs have formerly been proposed as a treatment for hard-to-heal and chronic wounds, leading to an improvement of healthy granulation tissue formation and wound area reduction, by ’kick-starting’ the healing process [18].

Therefore, the main objective of this work was to demonstrate that specific cell-to-NCM interactions prompt essential steps in the tissue regeneration and wound healing processes. For that, we assessed the biocompatibility of NCMs with the human cell types involved in the wound healing processes, their cell-adhesive properties and ability to induce proliferative responses, as well as their capacity to enhance macrophage switching from pro-inflammatory to anti-inflammatory phenotypes.

## 2. Materials and Methods

### 2.1. NCM Samples

All experiments were carried out using NCMs extracted from a commercial formulation (PolyHeal^®^ Micro, Madrid, Spain), consisting of polystyrene microspheres [4.5 × 10^6^ microspheres (MS)/mL] suspended in 22% glycerol and phosphate buffer (KH_2_PO_4_/Na_2_HPO_4_) in water for injection. Taking into account that a 22% glycerol concentration is toxic for cells in culture (due to the high osmolarity), the product was washed with _dd_H_2_O, centrifuged at 24,000× *g* for 10 min and opportunely resuspended. NCMs were counted by means of a TC20 automated cell counter (Bio-Rad, Madrid, Spain), and diluted as needed. The size of the purified NCMs was measured by means of dynamic light scattering (4.5 ± 0.2 µm) and their zeta-potential was determined through laser doppler micro-electrophoresis (−43 ± 1 mV). To keep the effect of glycerol constant, all assayed doses (NCM/cell) were prepared maintaining a final glycerol concentration of 0.44% (dilution 1:50).

### 2.2. Cell Culture

HaCaT keratinocytes (ATCC^®^, Manassas, VA, USA) were cultured in complete medium [Dulbecco’s modified Eagle’s medium (DMEM) (41965-039, Gibco^®^, MA, USA) supplemented with 10% (v/v) fetal bovine serum (FBS) and 1% (*v*/*v*) penicillin-streptomycin (P/S)]. Depending on the assay, other mediums were used such as assay medium (DMEM supplemented with 2% FBS) and starving medium (DMEM supplemented with 0.2% FBS). Cells were assayed between passages 3 and 5. 

Primary human dermal fibroblasts isolated from adult skin (HDFa, ATCC^®^, Manassas, VA, USA) were cultured on complete medium that consisted of fibroblast basal medium (PCS-201-030, ATCC^®^, Manassas, VA, USA) supplemented with a fibroblast growth kit-low serum (PCS-201-041, ATCC^®^, Manassas, VA, USA) and 1% P/S. Depending on the assay, other mediums were used such as assay medium (1:5 of complete medium in fibroblast basal medium) and starving medium (fibroblast basal medium). Cells were assayed between passages 3 and 5.

Human macrophages were obtained by primary monocyte isolation and differentiation from blood samples of healthy volunteers according to the ethical guidelines established by the institutional committee of the University of the Basque Country (UPV/EHU) (M30_2019_203). Peripheral blood mononuclear cells (PBMCs) were separated by Ficoll-Paque density gradient centrifugation. Then, monocytes were magnetically isolated using CD14 monoclonal antibody and cultured with complete medium (RPMI-1640, ATCC^®^, Manassas, VA, USA) supplemented with 10% FBS and 0.1% macrophage colony-stimulating factor (M-CSF) (Sigma-Aldrich, Química SL, Madrid, Spain) for 7 days in order to differentiate to M0 macrophages. Media was replaced every 2 to 3 days. Differentiation of M0 to M1 was induced by cultivating cells with 20 ng/mL of interferon gamma (IFN-γ) (Sigma-Aldrich, Química SL, Madrid, Spain) and 100 ng/mL of lipopolysaccharides (LPS) (Sigma-Aldrich, Química SL, Madrid, Spain) for 48 h. Similarly, differentiation from M0 to M2 was induced with 20 ng/mL interleukin 4 (IL-4). The M0 were maintained with M-CSF 0.1%. Success of the differentiation process was demonstrated by flow cytometry and optical microscope images (Figure S1).

Cells were incubated in a humidified incubator at 37 °C with a 5% CO_2_ atmosphere and cell passages were carried out every 2 to 3 days depending on the confluence.

### 2.3. Cytotoxicity Assay

Cytotoxicity assays were performed following the ISO 10993-5:2009 guidelines for biological evaluation of medical devices.

HaCaT and HDFa (5000 cells/well) and M0 macrophages (20,000 cells/well) were seeded into a 96-well plate on modified culture media supplemented with FBS. All plates were incubated for 1 h in 5% CO_2_ at 37 °C to allow complete cell attachment and stretching. Next, different NCM concentrations (0.1–200 NCM/cell) were added and incubated for 48 h. Dimethyl sulfoxide (DMSO, 10%), was used as cytotoxicity control. After 48 h, NCMs were removed from cultures and CCK-8 reagent (Sigma-Aldrich, Saint Louise, MO, USA) added and incubated for 4 h. Absorbance was read with a plate reader (Infinite^®^ 200 PRO series, Tecan Trading AG, Männedorf, Switzerland) at 450 nm and at 650 nm, as the reference wavelength. Based on these results, the NCM/cell ratios were established for further experiments.

### 2.4. Attachment Assay

NCM attachment on HaCaT and HDFa was evaluated through the fluorescent method described below. Fluoresbrite yellow-green NCMs (FYG-NCMs) (Polysciences, Inc., Warrington, PA, USA) were used. Briefly, 2 × 10^3^ cells/100 µL were seeded per well and cultured with complete medium for 24 h. Then, FYG-NCMs were added to render a final concentration of 50 FYG-NCM/cell. Fluorescence micrographs were taken with an epi-fluorescence microscope Nikon Eclipse TE2000-S (Izasa S.A, Barcelona, Spain) at different incubation times, and images were further analyzed with ImageJ. As cells retain the characteristics of their source tissue—single cells in close proximity (HDFa) or well-differentiated and spatially separated cell clusters (HaCaT)—, analyses were optimized in function of their culture particularities:

(i) For HaCaT, the attachment capacity of FYG-NCMs was semiquantitatively assessed by calculating the ratio between the fluorescence intensity within the area delimited by a cluster of cells and its bordering area (≈ 50 µm width).
(ii) For HDFa, adhesion of FYG-NCMs was semiquantitatively determined by calculating the particle aggregation factor, namely the average number of FYG-NCMs per aggregate. Briefly, fluorescence intensities obtained from areas with a known number of FYG-NCMs were used to calculate the average fluorescence intensity of a single FYG-NCM (at least 8 fields). Based on these data, the fluorescence intensities of at least 7 blindly taken fields were normalized to find the particle aggregation factor. Only fields with evenly distributed cells were included in the analysis.

For both cell types, the cell-adherence of the FYG-NCMs was assessed by comparing the results obtained at 0 h and 24 h (time required to allow completion of the cell adhesion process).

### 2.5. Proliferation and Viability Assays 

HaCaT and HDFa proliferation was assessed by BrdU. Briefly, 2 × 10^3^ HaCaT or 1 × 10^3^ HDFa cells were seeded per well. Prior to treatment, all HaCaT groups were cultured with complete medium for 6 h and then starved overnight. Subsequently, 50 NCM/cell and 10 NCM/cell were added and incubated for 24, 48 and 72 h. For each time point, a control group of 0 NCM/cell was used as reference.

In the case of the HDFa cells, all groups were cultured with complete medium for 24 h and then starved overnight. The starving medium was removed and replaced with assay medium to render a final concentration of 50 NCM/cell and 10 NCM/cell. Finally, plates were incubated for 24, 48 and 72 h. For each time point, a control group of 0 NCM/cell was used as reference.

All cells were cultured in the presence of BrdU (10 µM final concentration) for the last 2 h of each incubation time. All groups were assayed for BrdU uptake employing the cell proliferation Biotrak ELISA system (Amersham, NJ, USA), after complete removal of NCMs and following manufacturer’s indications. Absorbances (450 nm) measured for the nonspecific binding control group (technical control, without BrdU) were subtracted from all assayed groups, and the results of the treated cells were normalized against the corresponding control group for each time point.

Viability studies were run in parallel to the cell proliferation assays. Cells were washed twice with PBS and then dyed with the live/dead kit (Invitrogen, Thermofisher, Bilbao, Spain) following manufacturer’s instructions. After 30 min, fluorescence micrographs were taken with an epi-fluorescence microscope Nikon Eclipse TE2000-S (Izasa S.A, Barcelona, Spain).

### 2.6. Macrophage Phagocytosis Determination

The M0, M1 and M2 macrophages were incubated with 5 and 10 NCM/cell for 48 h and observed under the inverted contrast phase microscope Nikon Eclipse TE2000-S (Izasa S.A, Barcelona, Spain). In addition, the M0 macrophages were seeded on a sterile cover slip introduced in each well of a 24-well plate. After 1 h incubation, 5 NCM/cell were added and cells cultured for 48 h at 37 °C and 5% CO_2_. Cover slips were then washed with PBS and cells fixed in glutaraldehyde 2% for scanning electron microscopy (SEM) examination with a Hitachi S-4800 (Hitachi High-Technologies Corporation, Tokyo, Japan). For this purpose, samples were dehydrated and sputtered with gold.

### 2.7. Macrophage Polarization Assay

The ability of the NCMs to induce changes in the surface receptors of macrophages was evaluated by flow cytometry. The ability of the M0 macrophages to develop an anti-inflammatory profile, and the capacity of the M1 polarized macrophages to revert to an anti-inflammatory phenotype was analyzed. Cells were incubated with NCMs at 5 and 10 NCM/cell ratios for 48 h at 37 °C and 5% CO_2_. Next, cells were washed with PBS + 2 mM EDTA + 0.5% BSA, detached with TripLe™ and counted. 100,000 M0 or M1 were added to flow cytometry tubes. After Fc receptor blockade, cells were stained with anti CD14, CD64, CD83 and CD206 fluorescent antibodies, washed and analyzed by multicolor flow cytometry (MACSQuant 10, Miltenyi Biotec, Madrid, Spain). Macrophages were gated according to their forward- and side-scatter characteristics and data were analyzed by MACSQuantify software (Miltenyi Biotec, Bergisch Gladbach, Germany).

### 2.8. Statistical Analysis

Results are expressed as mean ± SD and all the experiments were performed at least in triplicate. Unpaired, two-tailed *t*-tests or Mann-Whitney *U*-tests were used for the attachment assay after testing for normal distribution. For the remaining experiments, differences among the groups at significance levels of 95% were calculated by ANOVA with Bonferroni or Tamhane multiple comparison corrections in function of their equality of variances. Statistical analysis was completed with IBM SPSS Statistics 20 program (SPSS Inc.^®^, Chicago, IL, USA).

## 3. Results

### 3.1. Cytotoxicity Study

NCMs were not cytotoxic in most of the studied concentrations (metabolic activity ≥ 100% as compared to the control). Only high concentrations of NCMs (over 100, 75 and 25 for HaCat, HDFa and macrophages, respectively) gave a viability under 100%. The appropriate NCM/cell ratio for further experiments was determined according to these results and the highest concentration that provided a viability greater or equal to 100% was selected. In addition, a lower concentration was chosen in order to determine the NCM dose-dependent effect. Thus, 50 and 10 NCM/cell ratio were chosen for HaCaT and HDFa, and 10 and 5 NCM/cell in the case of macrophages (Figure S2).

### 3.2. Attachment Assay

**Figure 1:**
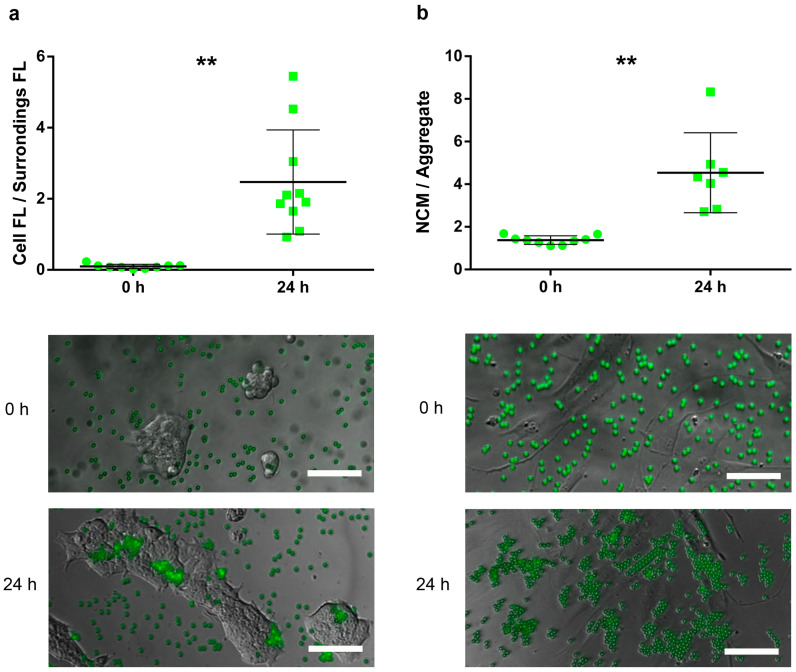
Attachment assay using fluoresbrite yellow-green negatively charged microspheres (FYG-NCMs) in HaCaT and HDFa. (**a**) Ratio between the fluorescence intensity within cell area and immediate surroundings assessed in the HaCaT cell line; FYG-NCMs suspended in _dd_H_2_O and incubated for 0 and 24 h; (**b**) Particle aggregation factor measured in human fibroblasts; FYG-NCMs suspended in _dd_H_2_O and incubated for 0 and 24 h; (**a–b**) Representative epi-fluorescence images of cells assayed with FYG-NCMs (green). **, *p* < 0.01. Scale bars, 100 µm. FL, fluorescence.

We assessed the cell-adhesive properties of NCMs in both HaCaT and HDFa. To visualize particle adhesion and obtain an accurate quantification, we used fluoresbrite yellow-green NCMs (FYG-NCMs) at 50 FYG-NCM/cell combined with image analysis. Freshly administered FYG-NCMs appeared in the close proximities of HaCaT cell clusters, with few particles within the area occupied by the cells (Figure 1a). After 24 h of incubation, we could observe how fluorescent particles reached the keratinocyte clusters and started to accumulate therein, forming aggregates onto the surface of the cell clusters (*p* = 0.001).

On the other hand, FYG-NCMs showed similar behavior when assayed in HDFa. At 0 h, fluorescence micrographs presented single and evenly distributed particles (particle aggregation factor = 1.38), which formed aggregates adapted to the cell shape after 24 h of incubation (Figure 1b). This gave, as a result, a statistically significant increase in the particle aggregation factor (*p* = 0.005). The NCMs remained adsorbed on the cell surface and no particle uptake was observed.

### 3.3. Proliferation and Viability Assays

**Figure 2:**
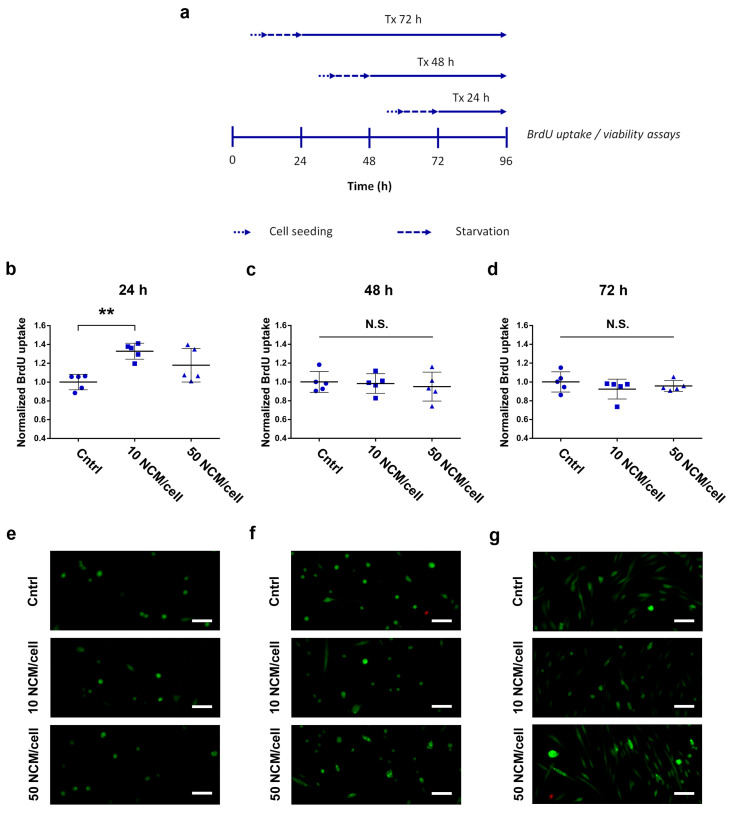
NCM proliferative response in HDFa. (**a**) Experimental design to assess BrdU uptake (**b**–**d**) and cell viability (**e**–**g**) after treatment with NCMs at different doses and exposure times. (**b**–**d**) BrdU uptake in human fibroblasts after 24 h (**b**), 48 h (**c**) and 72 h (**d**) of treatment; (**e**–**g**) Representative confocal fluorescence images of cells probed with the live/dead viability kit (green, living cells; red, dead cells) 24 h (**e**), 48 h (**f**) and 72 h (**g**) after treatment with NCMs. **, *p* < 0.01; N.S., nonsignificant (*p* > 0.05). Scale bars, 45 µm. Cntrl, control with no NCM exposure.

We evaluated the capability of the NCMs to promote cell proliferation in HDFa. We assayed the DNA synthesis rate and viability after incubation with 50 MS/cell and 10 MS/cell for increasing exposure times (Figure 2a). A statistically significant increase in the proliferation rate of HDFa was observed after 24 h of treatment with the lowest dose of NCMs (*p* = 0.001), while the highest dose showed nonsignificant results (Figure 2b). From this point onwards, treatments for both 48 and 72 h expressed nonsignificant differences against their corresponding control group (untreated) at any dose (Figure 2d). Fluorescence micrographs with calcein/ethidium dyes, taken in parallel to the proliferation assays, confirmed the compatibility of NCMs during the whole experiment, even at high doses (Figure 2e–g).

**Figure 3:**
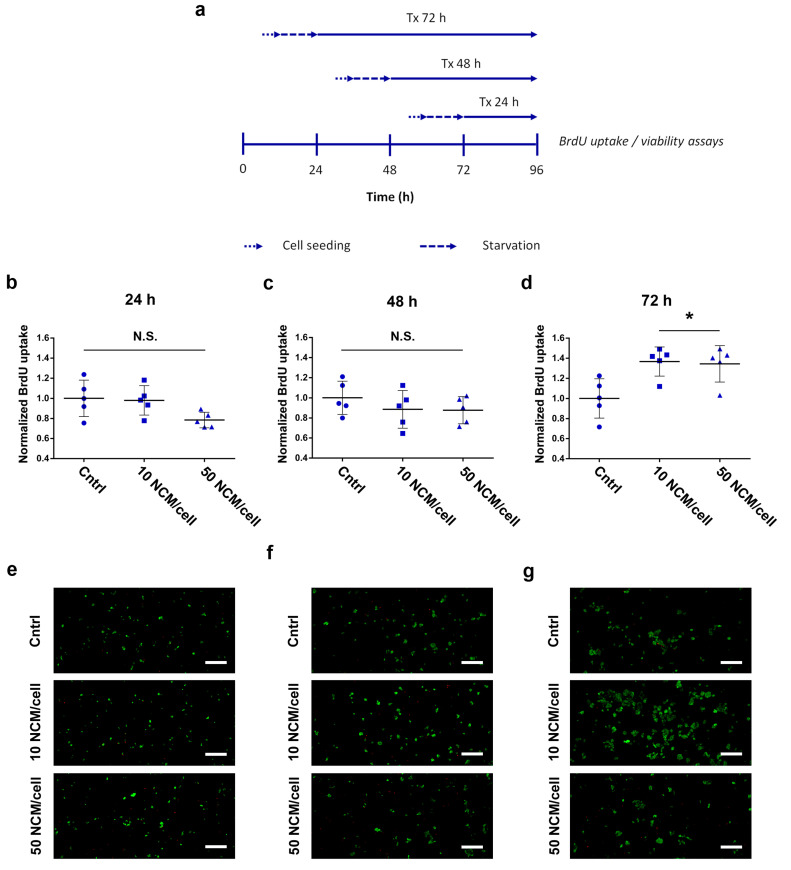
NCM proliferative response in HaCaT. (**a**) Experimental design to assess BrdU uptake (**b**–**d**) and cell viability (**e**–**g**) after treatment with NCMs at different doses and exposure times; (**b**–**d**) BrdU uptake in HaCaT cells after 24 h (**b**), 48 h (**c**) and 72 h (**d**) of treatment; (**e**–**g**) Representative confocal fluorescence images of cells probed with the live/dead viability kit (Green, living cells; Red, dead cells) 24 h (**e**), 48 h (**f**) and 72 h (**g**) after treatment with NCMs.*, *p* < 0.05; N.S., nonsignificant (*p* > 0.05). Scale bars, 200 μm. Cntrl, control with no NCM exposure.

We also assessed the capacity of NCMs to induce cell proliferation in HaCaT. We exposed keratinocytes to 50 MS/cell and 10 MS/cell doses for increasing incubation times, comparing their DNA synthesis rate and viability at each time point (Figure 3a). Treatments for both 24 and 48 h showed nonsignificant differences against their corresponding control group (untreated) at any dose (Figure 3b,c). Contrarily, we observed a statistically significant increase in cell proliferation for HaCaT cells after 72 h of treatment with both high and low doses of NCMs (*p* = 0.027 and 0.018 respectively) (Figure 3d). Fluorescence micrographs with calcein/ethidium dyes, taken in parallel to the proliferation assays, confirmed the compatibility of NCMs during the whole experiment, even at high doses (Figure 3e–g).

### 3.4. Phagocytosis Determination

The M0, M1 and M2 macrophages were observed under inverted contrast phase microscope after 48 h of incubation with 5 and 10 NCM/cells to determine NCM interaction with macrophage subsets. As shown in Figure S3, NCMs were always located in the area occupied by macrophages, and not in their surrounding area.

**Figure 4:**
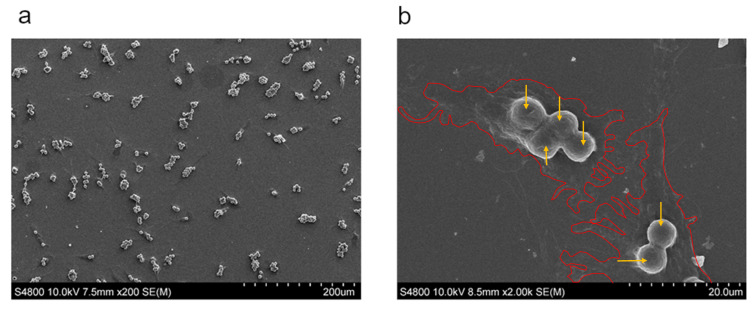
SEM images showing M0 macrophage after incubation with 5 NCM/cell. NCMs are colocalized with macrophages. (**a**) ×200; (**b**) ×2000. Edges of the cells (red line) and NCM (orange arrows) are shown.

To further confirm that NCMs were located inside cells, M0 macrophages were observed under SEM. The experiment was performed using 5 NCM/cell in order to facilitate visualization. Results showed a perfect colocalization of NCMs with macrophages (Figure 4a), and none located in the external surface. In addition, the cell membrane surrounding the NCMs could also be shown (Figure 4b), suggesting that they had been internalized by the cells. This fact was further confirmed through flow cytometry since an increase in cell complexity was found (Figure S4).

### 3.5. Polarization Assay

**Figure 5:**
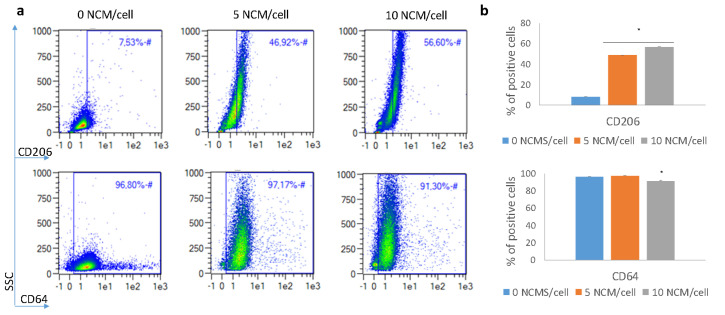
Surface marker changes when M0 macrophages were incubated with 5 and 10 NCM/cell. (**a**) Representative flow cytometry density plots. (**b**) Percentage of positive cells for each marker. *p < 0.05.

**Figure 6:**
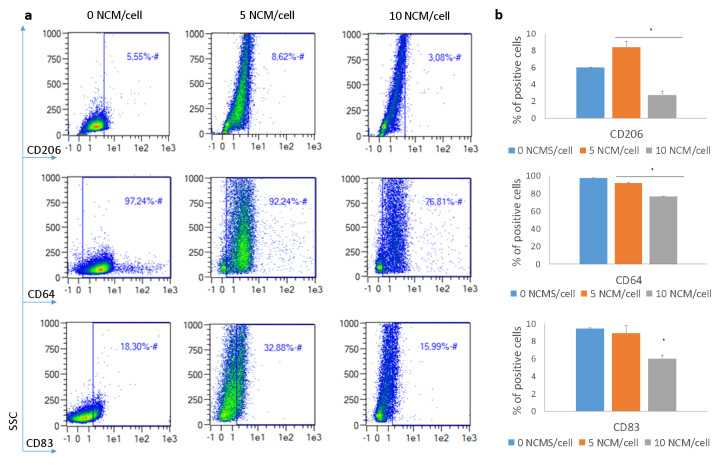
Surface marker changes when M1 macrophages were incubated with 5 and 10 NCM/cell. (**a**) Representative flow cytometry density plots. (**b**) Percentage of positive cells for each marker. *p < 0.05.

The ability of NCMs to bias macrophages to a noninflammatory stage was assessed by flow cytometry. Macrophages were identified based on forward and side scatter (FSC and SSC, respectively) and CD14 presence. Analysis of the macrophage surface-marker expression after 48 h of incubation with NCMs is shown in Figure 5 and Figure 6 for the M0 and M1 macrophages, respectively. Results showed that in the M0 macrophages, CD206 (typical M2 marker) was significantly upregulated, and CD64 (typical M1 marker) was downregulated, especially when the highest concentration of NCMs was used. Similar results were obtained for the M1 macrophages, and changes in the surface markers were even more pronounced in these cells. In this case, the expression of CD83 was also evaluated, showing a downregulation at the highest dose of NCMs. In most cases, changes in the surface-marker expression were dose-dependent, with the 10 NCM/cell ratio being the concentration able to induce the highest changes.

## 4. Discussion

In this work, we evaluated the potential of NCMs to enhance the wound-healing processes in terms of (i) compatibility of NCMs with relevant skin cells, (ii) their cell adhesive properties, (iii) their capacity to induce proliferative responses in target cells and (iv) their ability to switch macrophages to a noninflammatory stage.

Results from the cytotoxicity assay demonstrated good cell viability for most NCM concentrations tested, indicating their biocompatibility. However, at the highest NCM concentrations (≥ 100, 75 and 25 NCM/cell for HaCat, HDFa and macrophages, respectively) cell viability decreased. The selected NCM concentrations for further studies were 50 and 10 NCM/cell for HaCat and HDFa, and 10 and 5 NCM/cell for macrophages.

FYG-NCMs showed cell-adhesive properties in both HaCaT and HDFa. On the one hand, polystyrene has already been demonstrated to possess physical-chemical properties suitable for cell attachment, migration, proliferation and differentiation, since it has been specifically tested as a material for wound healing [19,20], among others. On the other hand, it is well-known that polymers are a cornerstone of regenerative medicine, directing interactions at the tissue–material interface [21]. Cell attachment to NCMs can be of noteworthy importance for the mechanism of action of the NCMs, since that contact may trigger cell proliferation and thus, wound healing [22]. In fact, studies suggest that the in vivo efficacy of wound-healing products can be directly related to their ability to be adhered to cells [23,24,25].

The NCMs demonstrated their capability to induce an improved proliferative response in the two cell lines studied. We subjected cells treated with NCMs to BrdU pulses of 2 h after the different incubation times with the aim of scanning when (or if) proliferative responses were promoted by NCMs. HDFa showed an increased DNA synthesis after 24 h of treatment at low doses (10 NCM/cell). The inhibitory effect, observed when too many adhesion molecules are present, is in agreement with previous studies carried out by either the current authors or other groups when working with 3D cultures modified with increasing concentrations of cell-adhesion ligands. These studies argued that the strong adhesion forces derived from an excessive number of bound receptors might hinder cell division [26,27,28,29]. On the other hand, HaCaT cells presented a delayed response, requiring 72 h of treatment, after which we observed a significantly increased cell proliferation both at high and low doses (50 and 10 NCM/cell). These results are in agreement with previous findings demonstrating that NCMs influenced the activity and proliferation of various cell types normally present in wounds [17]. It seems that NCMs’ specific surface area and geometry allow their interaction with cells through various mechanisms. First, exposure of NCMs to a biological environment results in the rapid adsorption of proteins to its surface [15]. In the same way, fibroblasts and keratinocytes deposit extracellular matrix-forming proteins (e.g., fibronectin, laminin and vitronectin), which are also adsorbed by NCMs and play a pivotal role in cell attachment [17,30]. We hypothesize that adsorbed proteins may interact with cellular receptors, especially with integrins; thus, triggering the activation of particular signal transduction pathways involved in cellular processes such as proliferation or migration [31,32]. Therefore, the nature and extent of this biologic response will largely depend on the integrin profile expressed by each particular cell type [33] and the layer of adsorbed proteins presented by the biomaterial surface [34]. In addition, further still-unraveled mechanisms may be responsible for cell attachment and subsequent biologic responses [35].

Regarding macrophages, we suggest that they are probably taken up by NCMs. This fact could be critical for the NCM effect, since particle accumulation in macrophages is thought to be advantageous for switching cell phenotype [36].

The polarization assay confirmed that NCMs may influence a macrophage’s phenotype and bias it towards a noninflammatory state. This, in turn, can lead to resolution of the inflammation process. In both M0 and M1 macrophages, the expression of CD206 marker was increased, a typical M2 marker indicative of the resolution of the inflammation [11]. Similarly, CD64 was downregulated, especially when the higher concentration of NCMs was used, in accordance with its lower expression in M2 macrophages [37]. In addition, the expression of CD83 was also assessed in M1 macrophages. Although this marker is not expressed in most macrophages, it can be found in M1 ones (as well as in mature dendritic cells) [38]. This marker was downregulated, especially when the highest dose of NCMs was used, indicating the loss of the M1 pro-inflammatory phenotype. Altogether, analysis of the surface-marker expression indicated that NCMs tended to favor a noninflammatory milieu, i.e., cells skewed their phenotype towards an M2 in the presence of NCMs. This fact may be important to explain the effectiveness of the treatment observed in humans [39]. However, the exact mechanism by which these NCMs skew macrophages towards M2 has not been determined. The most plausible explanation is that the biomaterial itself produces an immunomodulatory immune response, instead of activating the immune response. In fact, studies have demonstrated that macrophages in contact with several biomaterials produce lower levels of proinflammatory cytokines, such as IL-1β, CXCL8(IL-8), IL-12 and TNF-α, and higher levels of immunosuppressive cytokines, such as IL-10 [11,40,41]. Although the mechanism is not clear, NCM characteristics, such as physical properties (geometry, topography and porosity) and biochemical properties (surface chemistry, ligand functionalization and degradation mode) have been determined as critical factors in the host response to the material [42].

Thus, we can postulate that in vivo, NCMs mechanically promote (i) switching of macrophages towards M2 and (ii) the induction of fibroblast and keratinocyte proliferation.

With the aim of switching macrophage phenotype in chronic wounds, several approaches have been evaluated, including the use of mesenchymal stromal cells, growth factors, biomaterials, heme oxygenase-1 induction and oxygen therapy, among others [43]. Among biomaterials, several micro/nanoparticles have been evaluated, including or not bioactive molecules [44]. In the case of NCMs, no drug has been encapsulated; thus, the effects are directly attributed to the presence of NCMs on the wound. Similar studies have been carried out with chitosan microparticles [45] and although M0 to M2 polarization was observed, these microparticles were not able to suppress pro-inflammatory factor release from M1. Silica nanoparticles have also been evaluated with the same purpose [46]. In that study, the ability of the macrophage subset to phagocyte the microparticles was evaluated, but not the capacity to switch their phenotype, nor the effects for wound healing. Taking into account these results, our study demonstrated that NCMs possess several advantages in comparison to similar products, as they can promote M2 phenotype of macrophages overcoming the inflammatory stage, a common characteristic of hard-to-heal wounds.

## 5. Conclusions

We have proved that the mechanism of action of NCMs to promote wound healing is mainly caused by the capability of skin cells to interact with NCMs, which in turn induces cell proliferation and macrophage differentiation. NCMs showed excellent cell-adhesive properties in keratinocytes and human fibroblasts, and were able to promote a prompt response in the proliferative capacity of human fibroblasts after 24 h of treatment, and after 72 h in the keratinocyte HaCaT cell line. Importantly, NCMs seemed to be taken up by macrophages and were able to modify their surface-marker expression, so that pro-inflammatory macrophage populations could switch to a noninflammatory phenotype. This finding is of noteworthy importance since one of the main problems of chronic wound healing is the presence of classically activated macrophages that impair tissue repair. Thus, this ability of NCMs can be crucial to overcoming the inflammatory stage of chronic wounds, such as the diabetic wounds which are so challenging in clinical settings.

## Supplementary Materials

The following are available online at https://www.mdpi.com/2079-4991/10/6/1108/s1: Figure S1: Phase contrast images showing M0, M1 and M2 macrophage morphology, Figure S2: Cytotoxicity study for HaCaT, HDFa and macrophages after incubation with NCMs, Figure S3: Phase contrast images showing M0, M1 and M2 macrophage after 48 h of incubation with 5 and 10 NCM/cell, Figure S4: Representative density plots showing the different morphological features of M0, M1 and M2 macrophages when incubated with 5 and 10 NCM/cell.
