# Supplementary material for: Overcoming the Inflammatory Stage of Non-Healing Wounds: In Vitro Mechanism of Action of Negatively Charged Microspheres (NCMs)
Source: Nanomaterials (Basel). 2020 Jun 3;10(6):1108. doi: 10.3390/nano10061108 (PMC7353184; doi:10.3390/nano10061108)
Supplement: Supplementary file 1 [file nanomaterials-10-01108-s001.pdf]

nanomaterials

Supplementary Materials: Overcoming the Inflammatory Stage of Non-Healing Wounds: In Vitro Mechanism of Action of Negatively Charged Microspheres (NCMs)

Edorta Santos-Vizcaino, Aiala Salvador, Claudia Vairo, Manoli Igartua, Rosa Maria Hernandez, Luis Correa, Silvia Villullas and Garazi Gainza

M0

M1

M2

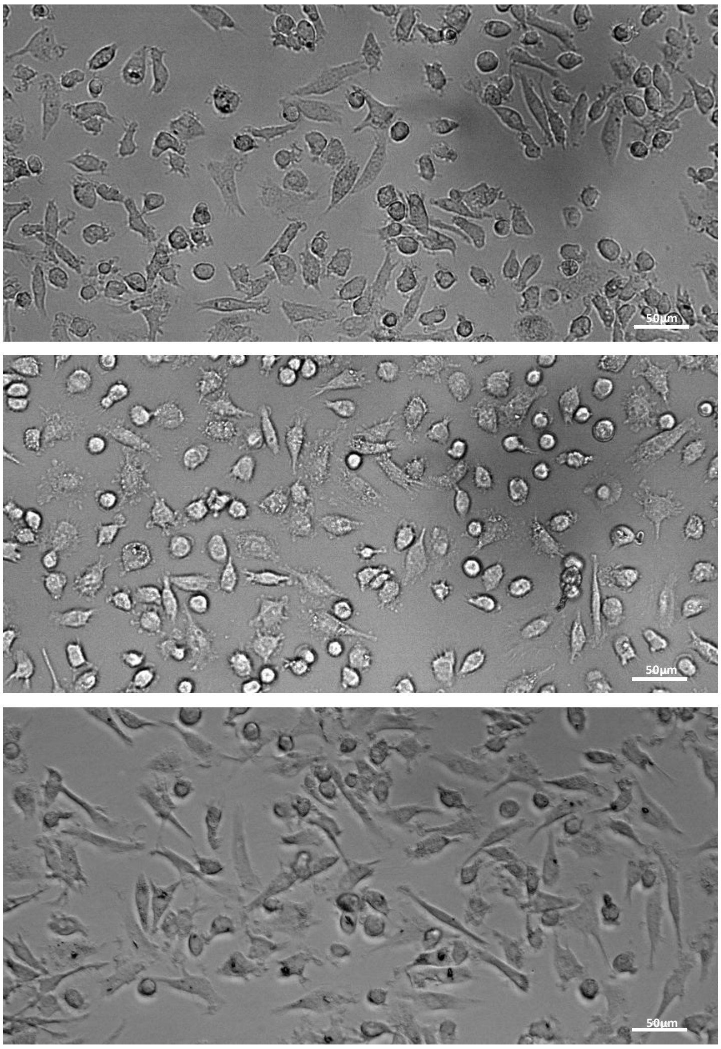

Figure S1. Phase contrast images showing M0, M1 and M2 macrophage morphology. As expected, M0 macrophages appeared elongated. Similarly, M2 had also an elongated shape, although some more rounded cells could be appreciated. On the other hand, M1 macrophages showed a dendritic morphology.

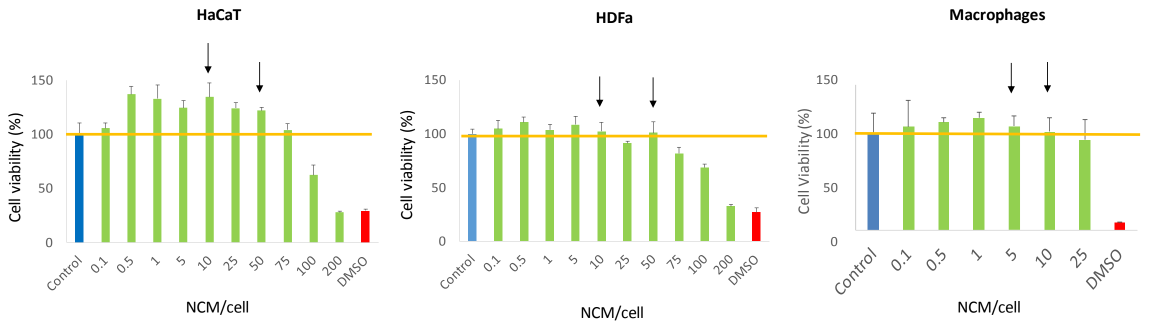

Figure S2. Cytotoxicity study for HaCaT, HDFa and macrophages after incubation with NCMs. Yellow line indicates 100 % viability. Arrows indicate concentrations selected for further studies.

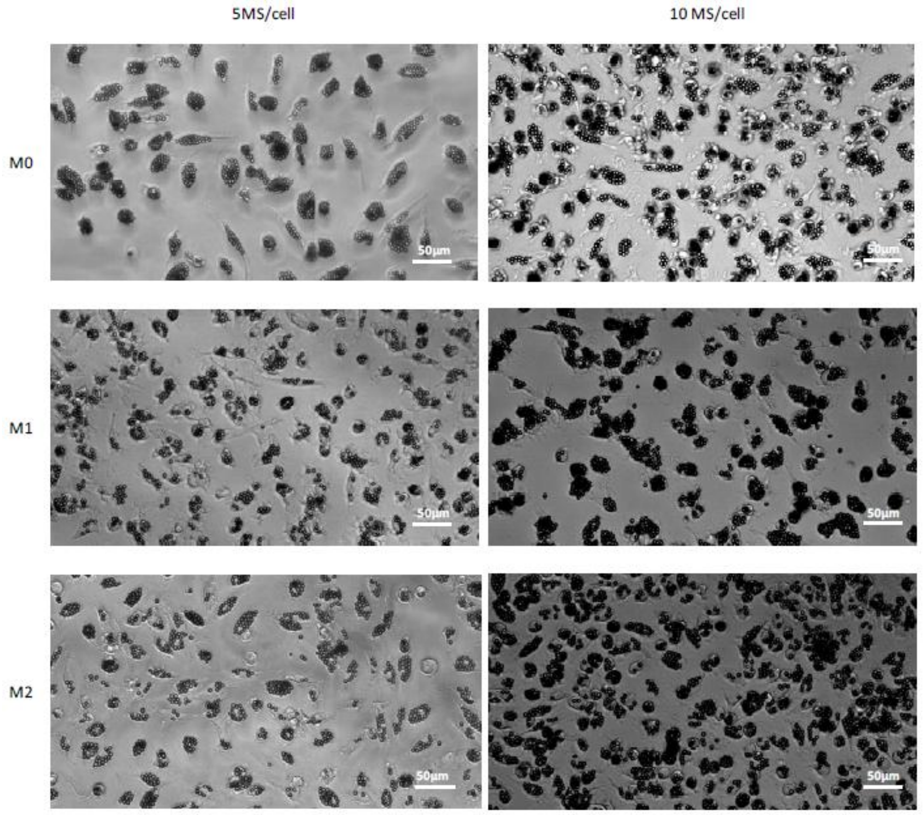

Figure S3. Phase contrast images showing M0, M1 and M2 macrophage after 48 h of incubation with 5 and 10 NCM/cell. NCMs can be observed in the same area occupied by the cells. Scale bars, 50 µm.

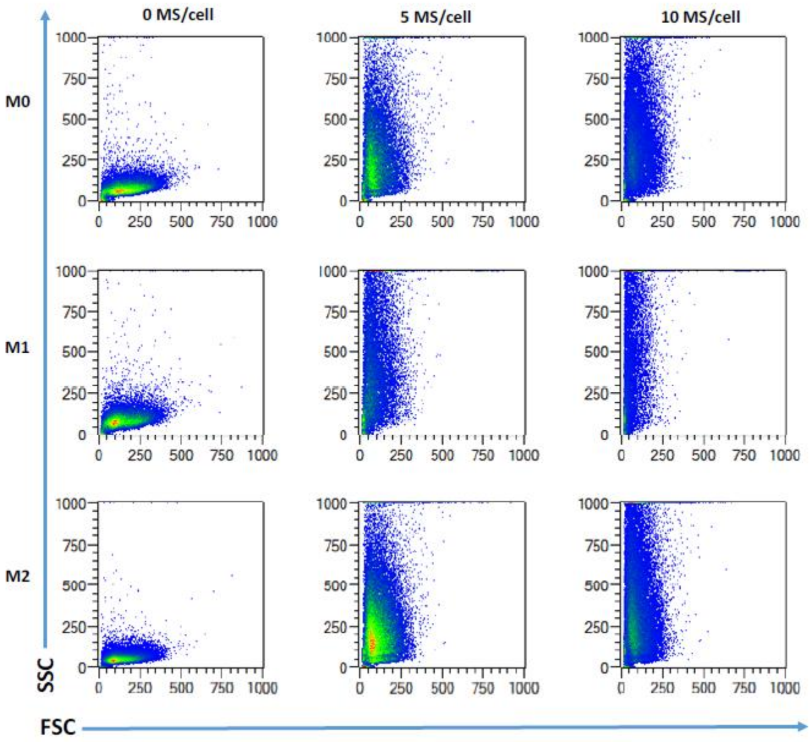

Figure S4. Representative density plots showing the different morphological features of M0, M1 and M2 macrophages when incubated with 5 and 10 NCM/cell. Increase in cell complexity indicates that cells have internalized the NCMs.
